# Remarks on the Physiological Action and Therapeutic Uses of Physostigma Venonosum, or the Ordeal Bean of Calabar

**Published:** 1873-12

**Authors:** J. Q. A. Hudson


### SOUTHERN MEDICAL RECORD.

VOL. III.—DECEMBER, 1873.—NO. 12.

### PART I.

### ORIGINAL COMMUNICATIONS.

### REMARKS ON THE PHYSIOLOGICAL ACTION AND THERAPEUTIC USES OF PHYSOSTIGMA VENONOSUM, OR THE ORDEAL BEAN OF CALABAR.

BY J. Q. A. HUDSON, M. D.

Read before the Meigs County, Ohio, Medical Association, November 3, 1873, and published by the request of the Association.

It is proposed in this paper, to present a brief summary of what is known up to the present time, of Physiological Action and Therapeutic Use of the Calabar Bean.

Its history may be briefly stated as follows:

The bean, the part of the plant in which the active virtues chiefly reside, was known to have been used by the savage tribes of the Gold Coast of Africa, as an ordeal test of guilt or innocence of those accused of certain crimes. An infusion of 19 seeds was administered, and unless vomiting occurred, death usually followed in about an hour.

Specimens of the bean were obtained from Africa by Dr. Christison, and planted in the botanical garden of Edinburgh; and subsequently, Dr. Balfour obtained the plant imported direct from Africa.

The plant received by Dr. Balfour and that raised from the bean by Christison were found to be identical. A botanical examination of the plant by Dr. Balfour, resulted in assigning to it a distinct genus, of which the plant is the only known species.

In 1863, Dr. Thos. R. Fraser, of Edinburgh, gave in an inaugural thesis, the results of his experiments and investigations, including what was then known, upon the calabar bean. From its physiological action, Dr. Fraser especially recommended a trial of the bean in tetanus and strychnia poisoning.

In 1867, Dr. Fraser, in the transactions of the Royal Society of Edingburgh, gave to the profession a second paper, more complete and full than the first, entitled, "On the Physiological Action of Physostigma Venonosum." The conclusions in this paper were drawn from numerous and varied experiments on animals. This second paper of Dr. Fraser, in the language of an eminent Professor, "is a model for this kind of investigation, and surpasses any thing that had hitherto been done in the way of the physiological study of remedies." (Prof. Bartholow.)

A third paper from the pen of Dr. Fraser, appeared in the transactions of the Royal Society of Edinburg in 1872, "On the antagonism between the action of Atropia, and Physostigma." This paper, like the second from Dr. Fraser's pen, is founded upon numerous and carefully executed experiments on animals, and its conclusions are well drawn.

It is but justice to Prof. Roberts Bartholow, to state in this connection, that to him is due the priority of establishing, by multiplied experiments, the antagonism between Atropia and Physostigma; which antagonism is fully proven with the experiments illustrating it, in his *Prize Essay on Atropia,* in the transactions of the American Medical Association for 1869, three years prior to the appearance of Dr. Fraser's paper. While we are ever ready to award to Dr. Fraser the greatest merit for his investigations upon physostigma and its uses, we must not forget what is due to our own equal, if not superior home talent, whose labors should have all the honor and distinction that rightfully belongs to it. It is undoubtedly true, that to Dr Fraser the profession is indebted for nearly all the knowledge we possess concerning the action of physostigma. The numerous reports in medical journals, and in the transactions of Medical societies, upon the uses of physostigma that have appeared since 1863—the date of Dr. Fraser's first paper—merely tend to confirm, in all important points, the conclusions of Dr. Fraser. I shall use the writings of Dr. Fraser freely, in this essay, when necessary to convey clear ideas of the action and uses of this agent.

I propose to treat the subject as follows :

1. The physiological action of physostigma upon the general system.

2. The physiological action of physostigma when applied to the conjunctiva.

3. The therapeutic uses of the agent in diseases affecting the general system, and

4. Its therapeutic uses in ophthalmic medicine and surgery, when applied locally to the conjunctiva.

Before proceeding to describe the systemic effects of the Calabar Bean, I wish to remark that the effect, when administered by the stomach, by subcutaneous injections, or by injection into the veins, upon the size of the pupil of the eye, is not uniformly the same. Prof. Roberts Bartholow, Hyp. Med., 2d edition, page 134, Dr. Thos. R. Frazer, American Journal of Medical Science, April, 1868, page 504, Prof. Geo. B. Wood, U. S. D., 13th edition, page 670, Dr. E. J. Waring, Prac. Thera., 2d American edition, page 447, each give as a constant result of the internal use of this agent, a contraction of the pupil. This contracted state of the pupil is probably nearly always observed in many animals and in man, when a large fatal dose is given. But this effect is rarely seen when a small fatal dose, or medicinal doses, are administered. In the majority of cases in which it has been administered in disease, the pupil is uninfluenced. In a small proportion of the cases, a contracted pupil has occurred.

Dr. J. T. Rothrock noticed in cats a dilitation of the pupil, resulting from its use. See Medical and Surgical Reporter, 1870, vol. 22, page 318.

M. Bourneville, a Paris Surgeon, states that "in nine cats and one dog, the dilitation was well marked and constant." In two cases of disease—one chorea, the other epilepsy—notwithstanding a gradual increase of the dose producing marked effects, he says, "The pupils never showed any contraction, but remained after the injections as they were before." See Half Yearly Compendium of Medical Science, January, 1870, page 46. In seven cases of chorea, under the care of Dr. W. H. Fuller, he states that the agent did not "materially affect the pupils." Braithwaite, January, 1872, page 65.

In two cases of the same disease, treated by Dr. John W. Ogle, he says "no change was observed in the pupils of the eyes." Braithwaite, January, 1866, page 82. Another case by Dr. Ogle, he says

"the pupils of the eye were rather enlarged." Braithwaite, July, 1866, page 71.

Dr. Ogle gives a history of a case of paralysis agitans, in which the patient took one and a half drachm doses of the tincture for two weeks, thrice daily, with no effect on the eye. Braithwaite, January, 1866, page 62.

In remarking upon a case of tetanus reported by Dr Eben Watson, (see Half Yearly Compendium of Medical Science, Jan., 1870, page 53) he says: "If this were a constant or even a common phenomena—contraction of the pupils—it would be an extremely useful guide in giving the bean, but that effect on the pupil is not often seen, unless the bean is applied to the eye itself." It is well to remember that Dr. Eben Watson has had more experience with the use of the bean in tetanus than any other single observer.

The foregoing statement of facts, will, I think, warrant the following conclusion, viz: That a contracted state of the ocular diaphragm is rarely seen as an effect of the calabar bean, except it be applied to the conjunctiva.

I have brought forward the foregoing facts to correct a wrong impression which the physician might receive from statements made in nearly all the leading text-books of the present time, in connection with systemic effects of calabar bean.

The effect of the calabar bean on the lower animals is quite similar, in all important points, to its effects on man. By reason of the many experiments made with it on animals, its effects on them are better known, and probably better understood, than its effects on man; yet its effects upon man, so far as observed, seem to be nearly the same. I shall quote from Dr. Fraser the effects produced on animals. He says:

"When a small fatal dose is administered to one of the lower animals, a train of symptoms is produced in the following order. A slight tremor is first seen, especially at the posterior regions, and this extends forwards to the anterior extremities and to the head. The limbs yield immediately afterwards, the posterior becoming generally first paralyzed, and the animal lies extended in a state of almost complete muscular flaccidity. A few attempts may be made to recover the normal position, but they are usually ineffectual. The bowels in most cases are evacuated, and urine is passed. The pupils generally contract; as the symptoms advance, respiration becomes slow and irregular, with a distinct stertor accompanying both inspiration and expiration; and frothy mucous escapes from the mouth. Muscular twitches occur, and often continue after respiration has ceased.

"Reflex action can not be produced, by either pinching or pricking the skin. By and by the eyelids do not contract when touched, or even the eyeball itself is pushed. On lifting by the ears, the limbs hang quietly, and the only sign of life is an occasional gasping inspiration, which also soon ceases, and the animal appears dead."

"Consciousness is preserved during the whole time until the power of expression is lost. During incomplete paralysis, proofs of sensation may be obtained by pinching the ears, or pricking the skin. Immediately after death the pupils dilate."

"When a large fatal dose of the kernel is administered, the hind limbs almost immediately yield, and the animal falls. It lies flaccid, and in any position on the table, and exhibits muscular power only by a few twitches. The pupils contract; in a few cases fluid escapes from the nostrils and mouth, and the lachrymal secretion is increased. Reflex action can not be produced by initation, and the respiration, after a few gasps, ceases. The pupils dilate immediately after death. On opening the body, muscular twitches occur. The heart is found distended and passive; irritation, however, produces contraction for about ten minutes after death. The vermicular action of the intestines is very much diminished, and can scarcely be observed.

"*Action on the Cerebrum.*—So far as paralysis is concerned, it is not produced by any action on the brain. Consciousness is preserved till death.

"*Action on the motor or efferent Nerves.*—In experiments frequently repeated, it was found that the motor nerves were not paralyzed before respiratory movements had ceased; although it occurred that the motor nerves were paralyzed immediately after the cessation of respiration.

"*Action on the afferent Nerves.*—The excitability of these nerves appears not to be affected, so long as the spinal cord retains its diastatic power. Instead of their excitability being diminished, it would appear to be actually increased.

"*Action on the Spinal Cord.*—The following experiment will show its power upon the spinal cord. A grain and a half of the extract, in fifteen minims of distilled water, was injected into the abdomen of a small dog. The animal sustained the paralytic shock, and in eleven minutes all respiratory movements had ceased. The spinal cord was immediately exposed, and the strongest galvanism, consistent with the localization of the current, applied to various portions of its substance, failed to excite any movements of the body. A sciatic nerve was then exposed, and slight stimulation of it produced vigorous contractions of the limbs, but no reflex movements. The heart continued to beat.

"*Action on the Heart.*—With very large lethal doses, the animal dies by cardiac syncope; with small doses, the heart beats are only diminished in power and pregnancy, and as the circulation continues, the spinal cord is more and more affected, until its diastaltic function is destroyed, and asphyxia is caused. From carefully conducted experiments with respect to the reduction of the heart's movements, the following summary may be presented: 1st. Diminution never preceded by increase of contraction, with no change of color on the occurrence of systole. 2d. Feebleness of contractions, with prolongation of the period of rest. 3d. Irregularity of rythm, the auricles contracting more frequently than the ventricles, and for intervals contracting alone. 4th. Stoppage of all the heart's chambers. If the poison be absorbed quickly, and in large quantity, the following fifth and sixth effects may not occur: 5th. Renewal of contraction by all the chambers at once, or by one or more in the first place. 6th. Gradual recovery to a low rate of action, and a continuance of this from a few moments to several days. 7th. Stoppage in diastole of spontaneous contractions. 8th. Loss of muscular irritability of the heart, rigor and change of reaction from alkaline to acid. Physostigma causes no acceleration, it diminishes the frequency of the contractions by prolonging the ventricular diastole, and produces a final stoppage by cessation of contraction of the ventricles, which themselves remain dilated, dark and full of blood.

The foregoing summary of the action of physostigma, which produced the effects described alike in warm as well as in cold blooded, animals, is taken from an analysis of Dr. Frazer's paper of 1867 made by Prof. Joseph Carson, of Philadelphia, and published in the American Journal of Medical Science, April, 1868, page 503 to 510.

I will now describe, as completely as my material will admit, the effects on man.

"Dr. Christison took 12 grains of the kernel, which, in fifteen minutes, produced giddiness and a feeling of torpidity, followed by great weakness and faintness, paleness of the surface, extreme weakness and irregularity of the pulse, and indisposition or inability to make voluntary muscular efforts. There was no pain or other uneasiness, except the feeling of prostration and some nausea, and the intellect was normal. In two hours after the poison was swallowed, drowsiness occurred, but no stupor. Dr. Fraser experienced from smaller doses effects of a similar character, with temporary dimness of vision. The heart appeared to be somewhat variously affected—sometimes acting irregularly or tumultuously, and sometimes less frequently. A peculiar epigastric sensation is generally experienced, as the first symptom, after the taking of the medicine, gradually increasing and becoming at lengh almost painful. This continues at intervals for a considerable time, is often a little while attended with some dyspnœa, and then dizziness and feebleness of the extremities are experienced." U. S. D., 13th edition, page 671.

In some of the recorded cases of tetanus, in which the calabar bean had been given in heroic doses, we have exhibited the effects of this agent.

In a case treated successfully by Dr. Eben Watson, it is stated that after using the bean in moderate doses for some days, with only a slight effect upon the disease, the patient, a girl of 11 years, was ordered half a grain of the extract every hour.

"By a mistake of the apothecary, the pills contained one grain instead of half a grain of the extract, and the girl had one grain every hour for nine hours; no particular effect being observed till half an hour after the ninth pill had been taken.

"The patient fell into the following state: Her eyes were widely opened, staring, and glassy; the pupils were contracted to pin points; the pulse was rapid and intermitting; there was a mucous rattle in the throat, and the breathing was jerky and fitful. Patient did not answer questions, and gave no signs of sensibility. She had no spasms, neither could they be induced. In fact, all the muscles were completely relaxed, except those of the back, which were still rigid. She either could not or would not move any of her limbs, or make voluntary efforts to swallow." See Braithwaite's Ret., July, 1867, p. 53.

By the use of brandy and water, and small doses of the tincture of belladonna, which was poured down the throat, the patient rallied and eventually recovered.

In a case of tetanus, treated successfully by Mr. Ashdown, the prompt effect of the remedy is well illustrated. One-third of a grain of the extract was used with the hypodermic syringe. The patient was a man 33 years of age. Mr. Ashdown says:

"The effect of the first subcutaneous injection was very marked.

In about five minutes the legs, which had been previously perfectly rigid and immovable, became flaccid and freely movable by the patient; the abdominal muscles became less tense, and the arching of the spine disappeared. The pupils also contracted, and the pulse sank to 82. The effects lasted between two and three hours, and all the symptoms then reappeared." Braithwaite, January, 1869, pp. 60-61.

There has been observed, in many of the cases in which the bean has been used, especially in those who have taken it in large and frequently-repeated doses, a profuse diaphoresis. This is not invariably seen, but it has been noticed so often, that observers are induced to believe it to be the effect of the remedy. It probably results from the extreme prostration which the agent produces.

In a certain proportion of cases there is vomiting and irritability of the stomach. Catharsis is often a result, and in nearly all cases the bowels respond mainly to medicine even in tetanus, in which disease constipation is often obstinate. We are now as well prepared as it is possible for us to be with the material in our possession, to present a summary of the action of calabar bean.

1st. It lessens the reflex action of the spinal cord, diminishing or destroying this function, according to the dose given. It is a perfect spinal paralyser. This is shown by the constant observable effects in small doses in man and animals, and it is beautifully illustrated by Dr. Fraser's experiment upon the dog, which being under the full influence of the poison, the cord did not respond to a powerful galvanic current, while the motor nerves responded promptly. See A. J. Med. Sci., April, 1868, p. 506.

2d. It acts in a slight degree to lessen the excitability of the motor nerves. Dr. Fraser is inclined to believe that the motor nerves are not influenced to any great extent, while the respiratory acts are performed, but they become paralyzed soon after respiration ceases.

3d. Muscular irritability is not affected.

4th. The excitability of the afferent nerves is not affected; their sensibility is sometimes increased.

5th. In small doses the action of the heart is weakened. This is shown, either by a lessening of the number of beats, or by an increase in the number of beats, with feebleness of action. In large lethal doses the action of the heart is at once destroyed, and death results from cardiac syncope. The action of the heart ceases in diastole.

6th. It sometimes contracts the pupil. This action probably frequently occurs with large doses, but when moderate doses are administered it is rarely observed. A dilatation of the pupil is an exceptional phenomenon.

7th. Catharsis is sometimes observed, and the same may be said of vomiting and diaphoresis.

The above items comprehend the chief points in the physiological action of physostigma, as at present understood. The action of calabar bean when applied to the eye, will now be considered.

In describing the peculiar action of calabar bean upon the eye, I cannot do better than to give the statement of the two leading Ophthalmological authorities of the present day, viz: Wells and Stellwag.

Stellwag says: "The calabar bean acts primarily and promptly upon the muscles of the iris, exciting contraction of the sphincter, and relaxation of the radiate fibres. If used to a large amount, it causes spastic contraction of the ciliary muscle. This is shown by the increase of the refractive power, even in eyes without an iris. These effects are confined to the eye to which the application is made. The pupil of the other eye is apt to enlarge on account of the diminution of the total impression of light acting on the two retinas." Stellwag's Discourse of the Eye, 2d American edition, page 25.

Wells says: "On the application of a strong solution, (of calabar bean,) 1 drop, equal to 4 grains, (of the powdered bean), to the inside of the lower eyelid, a little irritation and redness are produced, but these pass off very rapidly. Within five or ten minutes, the pupil begins to contract, and at nearly the same time the spasm of the ciliary muscle commences. The contraction of the pupil reaches its maximum degree in from 30 to 45 minutes. After two or three hours it gradually dilates again, but does not regain its normal size till after the lapse of two or three days, when it may even become larger than before. Even under its greatest contraction, the pupil is still under the influence of the light." Wells' Discourse of the Eye, 1st American edition, 1869, page 530.

In regard to its antagonism to atropia on the eye, he says:

"The effect of calabar bean in counteracting the action of atropia [on the eye,] has also been proved by many experiments. The weaker solutions of atropia are easily overcome by a strong solution of calabar. But the complete paralysis of accommodation by a strong solution of atropia, (4 gms. to the ℥,) is only temporarily overcome even by a very strong solution of calabar. (1 drop=4 gns.) The pupil becomes smaller, and the state of refraction increased; but the action of atropia reasserts itself in the course of a few hours. In such cases we must repeat the application of the calabar when necessary, until the effect of the atropia upon the accommodation has disappeared." See Well Ed. cited above, p. 530.

From the above statement by Wells, it appears that in the antagonism, atropia is the more powerful agent, the action of which is more lasting than that of the physostigma. Having now considered the physiological action of calabar bean upon the general system, and upon the eye, I will proceed to give the uses of the agent in diseases affecting the general system. The constant and prompt action of this agent upon the spinal cord, acting as a paralyzer, and destroying reflex action, points to its use in those diseases in which this function is in a morbidly excited state; and it is in these diseases that its use has been found most beneficial—particularly in tetanus and in poisoning by strychnia.

Strychnia poisoning, as is well known, produces a condition of the system that often can not be easily distinguished from tetanus. Dr. Fraser particularly recommended the calabar bean as a remedy worthy of trial in tetanus and strychnia poisoning. The remedy is of such great power that its internal use requires constant vigilance in observing its effects, that the patient be not overdosed and poisoned.

The difficulty and sometimes impossibility of securing attendants who are to take charge of the sick and administer the remedy, having judgment to guide them in the absence of the physician, is a hindrance that will sometimes prevent that free use of the remedy in tetanus that is generally essential to success. It is well known that the tetanic paroxysms are very exhausting and depressing to the system, and that the convulsions should, if possible, be speedily controlled and prevented.

In the majority of cases of tetanus, the calabar bean will either wholly control and prevent the tetanic paroxysms or it will lessen the frequency and severity of them, as well as shorten their duration. In many cases of tetanus in which this agent has been unsuccessfully used, it has been employed in a timid, imperfect manner, resulting in death to the sufferer, and disparagement to the remedy.

Those who have used this agent in tetanus are at one accord in their advice to commence with moderate doses, to be increased gradually, and given at comparatively short intervals until the system begins to exhibit the influence of the remedy, or until the disease begins to yield. This is especially essential in the acute and rapidly progressing form of the disease. The quantity of the agent required in each case can only be known upon trial.

In some severe cases, moderate doses will have a powerfully controlling effect; in other cases, equally severe, the remedy may have to be doubled or trebled in amount before the desired effect is reached. These large doses, according to the history of recorded cases, have been continued for days, and have been required, to keep the disease in check, as upon their withdrawal the spasms would return, to disappear upon the renewal of the medicine.

It may be said then, that each case of tetanus must be studied by itself, and by careful trial the dose determined that is required to hold the morbid action in abeyance, and then the medicine carefully continued during the progress of the disease, lessening the dose and gradually withdrawing the agent as the disease subsides.

The plan recommended and pursued by Dr. Frazer, is to commence the treatment by using the remedy by subcutaneous injections at intervals of one or two hours, until the disease yields, or the system is influenced, and then to continue the medicine by the stomach, in three times the quantity used by the syringe, every two or three hours, as may be requisite. He insists that in severe acute cases the remedy be commenced and continued by the hypodermic syringe. Every one knows the quickness and the certainty of the action of medicine by the hypodermic method, and a few hours of severe tetanic convulsions may terminate in the death of the patient. The evidences of the action of the physostigma are relaxation of the spasm; this is the principal effect. This may be to such an extent that as to relieve the muscles of deglutition, permitting the patient to swallow, when food of an easily digestible nature should be freely but judiciously given; and if evidences of debility exist, alcoholic stimulants in some form should be administered. Alimentation and stimulation form a prominent and important part of the treatment, and should be enforced during the whole course of the case. Another evidence of the action of the drug is the condition of the pulse. The pulse will be lessened in frequency and force. This is the usual indication of its action on the heart. In some cases a less desirable state of the pulse will be found, viz: an increase in the frequency, with great feebleness. This would signify a powerfully depressing influence on the circulation, and sometimes would point to a withdrawal of the remedy for a time. In a case treated by Mr. Ashtown, the pulse, under the influence of the bean, rose to 148. Its use was stopped 7 hours and it fell to 125.

Contraction of the pupil may or may not occur; should it appear, it may be a valuable aid to determine the effect of the bean on the system, according to its condition of contraction.

The following rules may be observed, in the use of the calibar bean in tetanus.

1st. Commence the treatment by the subcutaneous injection of $\frac{1}{4}$ or $\frac{1}{3}$ of a grain of the extract dissolved in water, repeating and increasing the dose according to its effect, every hour or two hours, till the disease yields, or the action of the remedy is exhibited.

2d. If the case is severe and spasms violent, continue the hypodermic method.

3d. If not severe, after the use of the hypodermic method in the start, give the remedy by the stomach every two or three hours, in treble the quantity used with the syringe.

4th. The patient must have a liberal supply of nourishing food and stimulation, (if symptoms of debility arise,) during the whole course of the disease.

5th. Should there be manifestations of increased excitability of the sensory nerves, the exhibition of morphine, hydrate of chloral, or bromide of potassium will aid in controlling this state of the sensory nerves.

The question arises naturally, "Are there not cases of tetanus over which physostigma has no control, and in which its use may be injurious, either directly or by taking the place of other remedies when it is administered, and it seems to have no control over the case?" This is an important query, and I find that it is nowhere discussed in any of the articles I find in Journals. There must be idiosyncrasies as regards the action of this medicine as there are in regard to the action of nearly every other medicinal agent used, and might there not exist in certain constitutions a peculiarity or peculiarities unknown, in which physostigma would have no action, or would have an action entirely different from that it usually exhibits?

We can not but admit that there do exist idiosyncrasies as to the action of calabar bean. There is another point. Any person reading over carefully Dr. Fraser, can not but conclude that physostigma has some action upon the sympathetic nervous system, but what that action is, is not precisely determined. This action on this system is more prominent in some persons than in others, since in some individuals we find vomiting and gastric irritability occurring; in others, there is extreme cardiac depression; and there are a few recorded examples in which the remedy has had a full and free exhibition in tetanus, with no control over the disease. My own crudely formed opinion upon this question is this: That if after the free exhibition of this remedy, it seemed to have no control over the spasmodic action, and especially if there should occur extreme cardiac depression, I should feel as though I ought to withdraw it, and use other agents that have at times succeeded in its cure. Should an obstinate irritability of the stomach arise, precluding the use of food, the exhibition of which is so important in tetanus, I should feel inclined to give up the agent, unless its control over the disease was very marked and certain.

Calabar bean has been used successfully in strychnia poisoning. It is the most perfect antagonist to the action of strychnia known. This was first demonstrated by Dr. Fraser in his experiments on animals, and has since been tested satisfactorily on the human subject.

The following case is recorded by Dr. Jno. W. Keyworth. I will give the case as reported by Dr. Keyworth, as it is very interesting.

"On the 23d of July, 1867, at 7 a. m., I was sent for to see a female patient of slight suicidal tendency, who had taken at 11 o'clock the night before, a packet of Battle's Vermin Killer, containing, it is stated, about 3 grains of strychnia. The body and limbs were perfectly rigid with spasms, was excited by merely blowing in her face, or by the shaking of the floor in walking. She had been in this condition since 2 a. m., gradually becoming worse. She was speechless, but conscious, and her pulse very rapid and feeble. Her face was livid; the jaws were constantly fixed. Having recently read your paper, and seeing also that the case was almost hopeless, I sent up to the eye infirmary for some of the wafers. Failing there, I obtained from Messrs. Southall, of this town, pharmaceutists of the first order, some tincture of calabar bean. They could not give me any information as to the dose. I gave 30 drops every half hour till two drachms had been taken, pouring it into the mouth where a tooth was absent. The effort of swallowing produced violent spasm. After each dose the attacks grew less frequent and strong, and I then gave two half drachm doses at intervals of an hour, then 15 drops every two hours. Altogether half an ounce was taken. The pulse then became extremely weak, the spasms almost ceased, but the woman could scarcely speak or swallow. At the end of some hours, she remained very feeble, quiet, but sleepless, and her legs and arms felt like lead, and were utterly immovable. This gradually subsided, but she could not stand or walk for four or five days. and was three weeks before she fully recovered. The effect of the calabar bean in relaxing the spasms was apparent in about twenty minutes after taking the first dose, and became more apparent and decided after each repetition. Glasgow Medical Journal, Nov. 1868, page 54. Quoted in Braithwaite, July 1873, pages 71 and 72.

It is very evident that in strychnia poisoning, as well as in tetanus, when the spasm of the muscles of the throat render swallowing difficult, and sometimes impossible, that the hypodermic use of the medicine is to be preferred.

Physostigma has been used in a few instances with success in atropia poisoning. A case is reported in the American Journal of Medical Science for April, 1865, p. 472, quoted from a Prussian journal, in which the calabar bean was used successfully. The poisonous action of the atropia was marked and violent. One dose of the calabar produced violent vomiting in fifteen minutes, followed by a rapid subsidence of the symptoms of atropia poisoning.

In the same article another case is given in which the atropia poisoning was slight, but yielded rapidly to the internal administration of the bean.

A third case is reported in the Half Yearly Compend of Medical Science for July, 1872, p. 46, quoted from the Australian Medical Gazette. A boy two and a half years of age was poisoned by taking internally by mistake some belladonna liniment. The 19th of a grain of the ext. phys. was given, with a prompt subsidence of the symptoms of belladonna poisoning, which were very marked and powerful.

The antagonistic action of opium to belladonna or atropia poisoning is, in the opinion of the writer, to be preferred to the use of physostigma. The physiological action of atropia has been carefully studied by many eminent men, and still we are far from comprehending its full extent of action. It is probably a stimulant and a powerful pain destroyer, but further than this, its powers and action are not understood. It is stated to be a spinal paralyzer, (Professor Bartholow,) but in the opinion of the writer this action is questionable. If it had this power, its use with physostigma, which is known to have this property, would still further depress the action of the cord, and produce dangerous or fatal results; but facts oppose this view. Atropia relieves the system from the poisonous action of calabar bean, without any increase at least in the condition of spinal paralysis. Dr. Fraser in his paper on the antagonism between atropia and physostigma, does not attempt to explain the cause of the antagonism, but merely by experiments gives the facts, with no exposition or theory. One point is shown by his experiments, viz: that a comparatively small dose of atropia will overcome the effects of a large dose of physostigma.

Calabar bean has been employed in the treatment of chorea. It was first employed by Dr. Jno. W. Ogle, who reports several cures. Other parties have not had the same success.

Dr. W. H. Fuller employed physostigma in seven consecutive choric patients, with negative results. M. Bourneville reports a case in which after a full trial there was no amendment. Other observers have published favorable results.

Dr. H. N. McLaurin publishes a case of cure. See Cin. Lan. & Obs., 1865, p. 768.

Dr. F. A. Ashford gives a case in which there was a decided amelioration, but the final result is not given. Half Yearly Comp. Med. Sci., Jan., 1872, pp. 123-124.

A case of chorea was cured in 1863 by Dr. George Harley. This is the first published case of its use in this disease.

Chorea is one of those diseases, the pathology of which is imperfectly known; and for the cure of which remedies are used in each case by way of experiment. If one medicine after a fair trial fails, another is employed; and it too often happens that the whole round of medication is employed, the disease remains the same.

The profession should be thankful for an accession to the list of remedies in chorea. Physostigma is added to the list. The success of the remedy will probably be the greatest in those cases in which there is an excited condition of the reflex action of the spinal cord.

*Epilepsy.*—In this disease Dr. S. W. D. Williams records the results of its use in the Sussex Lunatic Asylum, of which he was at the time Superintendent. The physostigma was administered to twelve epileptics, in doses of one grain of the powdered bean twice daily. He says:

"In six of the cases under treatment, there was a very considerable decrease in the number of fits during the six months they were taking the bean, as compared with the six months previously; and what is still more remarkable, that when the bean was omitted, the fits, without exception, began to increase in every case. This was very marked in some cases." Braithwaite, July, 1872, p. 87.

The above showing denotes that a further trial of physostigma in epilepsy is desirable.

Physostigma has been used to a limited extent in other diseases, as in obstinate constipation with fecal accumulation, which it relieved. See Southern Med. Record, Feb., 1873, p. 103. Also, in hemicrania. See same journal, Aug., 1873, p. 488. The extent of its uses will undoubtedly become greatly enlarged, by further trials of it in various diseases, and particularly when its action on the sympathetic system of nerves is better known.

I will close this part of my subject by quoting a statement by Dr. Fraser respecting the employment of physostigma in cases of disease exclusive of tetanus and strychnia poisoning. He says:

"I have been principally guided in my selection of cases by the condition of the pulse; a pulse in any wise feeble being considered a decided contra-indicator, while one that was strong, rapid and hard, was considered a true indicator for the employment of the drug. I have found this action, (viz: the sedative action on the heart,) of value in erysipelas, delirium tremens, febricula, acute bronchitis, and rheumatic fever, and have detailed a few cases from a number in which this treatment was tried."

It now remains for us to examine the therapeutic uses of the local action of calabar bean upon the eye, which I will do with as much brevity as is consistent with a clear understanding of the points involved.

1st. Calabar bean may be used to counteract the action of atropia.

The use of the strong solution, 1 drop—equals 4 grains—will be required when the action on the eye is the result of the stronger solutions of atropia. As the action of atropia is more persistent than that of the calabar, the latter will require to be supplied from time to time till the power of accommodation is restored.

2nd. In cases of prolapsus of the iris, from whatever cause, the use of calabar bean is useful to restore the iris and prevent prolapse. If the calabar bean is applied before adhesion of the prolapsed iris takes place, the prolapsion may be prevented, or if applied after adhesions have formed, the calabar will aid in breaking up the slighter adhesions, and preventing further prolapse. Numerous cases are reported in which its employment in prolapse, either with or without adhesion, has resulted in great benefit in restoring the parts.

3d. Previous to the operation of endoctomy the pupil should be placed under contraction by the calabar.

4th. In cases of idiopathic mydriasis, arising from essential palsy of the third nerve, its use is indicated to restore the nerve power. These cases are probably rare. A feebleness of nerve power sometimes results from debility following disease, as diphtheria, typhoid fever, &c. The use of the calabar will speedily overcome this paralysis from debility. But mydriasis arises from many other causes, in which its use would primarily be either injurious or useless. The physician should be careful in diagnosis. Such cases are mydriasis from rheumatic inflammation, syphilis, or from reflex action from distant organs, as the stomach, uterus, &c. It may also occur accompanying amaurosis. In some of these last named conditions, its use might be either nugatory or injurious, especially if employed before the use of other means to remove the diseased condition, after which, the disease upon which it depends being removed, should the power of accommodation not return, a gentle stimulation of the third nerve might be judiciously resorted to. There are other uses of the calabar bean in ophthalmology, that belong rather to the nicer discriminations of the specialist, which I shall not enter upon this paper.

*Preparations.*—The only official preparation of the U. S. P. is the alcoholic extract, (ext. phys.,) of which the dose is given at from 1-16 to 1-4 of a grain. The dose of the powdered kernel is given at 2 to 3 grains, to be increased if necessary.

In tetanus, as has been before stated, no rules of dosage can be given. The remedy should be administered until its effects are produced upon the system or the disease, and if necessary in rapidly increasing doses until these results are accomplished.

Dr. Fraser recommends that the treatment be commenced with the subcutaneous injection of ½ of a grain. In one case of tetanus by Dr. Bontflower, the patient took 90⅗ grains of the extract in 9 days, and part of this was used by subcutaneous injection. This is equal to over 10 grs. in 24 hours.

In a case reported by Dr. Eben Watson, the patient took, in 43 days, 1026 grains of the alcoholic extract, equal to an average of over 23 grains of the extract in 24 hours.

---

Butter-Milk in Typhoid Fever.—Dr. DeLisle has had great success in treating this fever with butter-milk, reducing the tympanitis, etc., and regards it as a prophylactic when taken largely when the disease is prevailing.

